# Acute invariant NKT cell activation triggers an immune response that drives prominent changes in iron homeostasis

**DOI:** 10.1038/s41598-020-78037-3

**Published:** 2020-12-03

**Authors:** Hua Huang, Vanessa Zuzarte-Luis, Gabriela Fragoso, Annie Calvé, Tuan Anh Hoang, Manon Oliero, Geneviève Chabot-Roy, Victor Mullins-Dansereau, Sylvie Lesage, Manuela M. Santos

**Affiliations:** 1 Centre de Recherche du Centre Hospitalier de l’Université de Montréal (CRCHUM), Montréal, Québec Canada; 2 Département de Médecine, Université de Montréal, Montréal, Québec Canada; 3 Instituto de Medicina Molecular (iMM), Lisbon, Portugal; 4 Département de Microbiologie, Infectiologie et Immunologie, Université de Montréal, Montréal, Québec Canada; 5 Maisonneuve-Rosemont Hospital Research Centre (CRHMR), Montréal, Québec Canada; 6 Nutrition and Microbiome Laboratory, CRCHUM-R10.426, 900 rue Saint-Denis, Montréal, Québec H2X 0A9 Canada; 7 Present Address: Centre INRS-Institut Armand-Frappier, Institut National de La Recherche Scientifique, 531 Boulevard des Prairies, Laval, Québec Canada

**Keywords:** Homeostasis, Acute inflammation

## Abstract

Iron homeostasis is an essential biological process that ensures the tissue distribution of iron for various cellular processes. As the major producer of hepcidin, the liver is central to the regulation of iron metabolism. The liver is also home to many immune cells, which upon activation may greatly impact iron metabolism. Here, we focus on the role of invariant natural killer T (iNKT) cells, a subset of T lymphocytes that, in mice, is most abundant in the liver. Activation of iNKT cells with the prototypical glycosphingolipid antigen, α-galactosylceramide, resulted in immune cell proliferation and biphasic changes in iron metabolism. This involved an early phase characterized by hypoferremia, hepcidin induction and ferroportin suppression, and a second phase associated with strong suppression of hepcidin despite elevated levels of circulating and tissue iron. We further show that these changes in iron metabolism are fully dependent on iNKT cell activation. Finally, we demonstrate that the biphasic regulation of hepcidin is independent of NK and Kupffer cells, and is initially driven by the STAT3 inflammatory pathway, whereas the second phase is regulated by repression of the BMP/SMAD signaling pathway. These findings indicate that iNKT activation and the resulting cell proliferation influence iron homeostasis.

## Introduction

Anaemia of chronic disease (ACD) or anaemia of inflammation is the second most prevalent cause of anaemia, after iron deficiency anaemia^1^. ACD is driven by the activation of immune cells and their production of inflammatory cytokines^1^. Hallmarks of ACD include disturbances in iron homeostasis that divert iron from the circulation into storage sites, thereby reducing iron availability for erythropoiesis^2^. Disrupted iron homeostasis in ACD is achieved via the regulation of genes involved in cellular iron uptake and export, and leads to increased uptake and retention of iron within cells of the reticuloendothelial system. Specifically, tumour necrosis factor alpha (TNF-α), interleukin (IL)-1, IL-6, and interferon gamma (IFN-γ) are cytokines that can directly modulate the translation/transcription of genes involved in iron homeostasis, such as the iron-regulatory hormone hepcidin in the liver^3^. The main cell types that produce these cytokines include T lymphocytes and macrophages, and their activation contributes to ACD^1,4^.

The liver is the major regulator of systemic iron homeostasis through hepcidin production^5,6^ and is home to many immune cell types^7^, including conventional and non-conventional T cells^8^. In mice, invariant natural killer T (iNKT) cells are most abundant in the liver. These iNKT cells express T cell receptors (TCR) that recognize lipid or glycolipid antigens presented by the MHC class I-related glycoprotein CD1d^9,10^. In the absence of effective lipid presentation by CD1d, iNKT cells accumulate in the liver and contribute to inflammation^11^. However, in the presence of obesity, presentation of endogenous lipids to iNKT cells in the liver also contributes to inflammation^12^. Indeed, activated iNKT cells rapidly secrete both Th1- and Th2-type cytokines^13^, which facilitates the recruitment and activation of many other immune cells, such as NK cells, myeloid cells, and various T cell subsets, to stimulate anti-tumor immune responses^14,15^. As such, the release of large amounts of cytokines by activated iNKT cells^13^ may potentially affect iron homeostasis by inducing hepcidin expression in the liver^16^. Thus, iNKT cells represent a unique cell population that may influence iron metabolism.

Here, we investigated the influence of iNKT cell activation on iron metabolism. Our results indicate that iNKT cell activation induces profound alterations in iron homeostasis.

## Results

### Activation of iNKT cells in vivo with α-GalCer induces changes in iron metabolism

**Figure 1:**
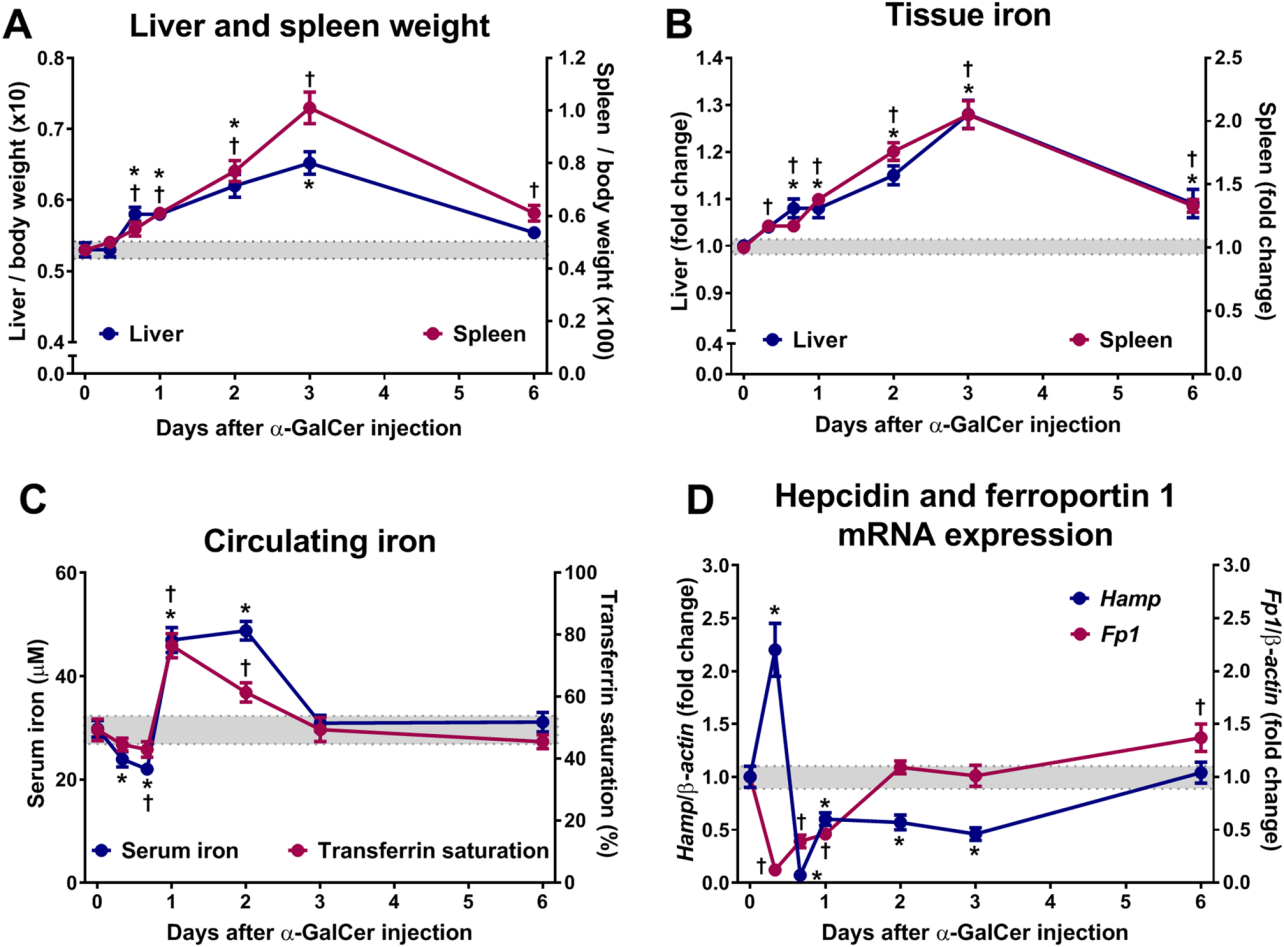
Time course of α-GalCer-induced changes in systemic iron metabolism. Wild-type mice were injected with vehicle (indicated by the gray area across the graphs) or 100 μg/Kg body weight of α-GalCer. (**A**) Liver and spleen weights. (**B**) Total iron in liver and spleen. (**C**) Serum iron and transferrin saturation. (**D**) Hepcidin (*Hamp*) and ferroportin 1 (*Fp1*) mRNA expression. Data are presented as mean ± SEM for a minimum of *n* = 12 mice per time point. Statistical analysis was performed with one-way ANOVA. *(blue lines) and ^†^(purple lines) *P* < 0.01, compared to mice injected with vehicle at each time point.

The prototypical iNKT cell ligand, α-galactosylceramide (α-GalCer), induces a potent primary iNKT cell response^17^. To evaluate the impact of acute iNKT cell activation on iron metabolism, we injected wild-type mice with α-GalCer and monitored the mice for six days. As expected, α-GalCer treatment led to a significant increase in liver and spleen weights, peaking at day 3 (Fig. 1A). This is in line with a robust iNKT cell activation, leading to immune cell recruitment and proliferation^18,19^. iNKT cell activation also impacted iron metabolism. Total iron levels in the liver and spleen increased, mirroring the increase in liver and spleen weights (Fig. 1B). In contrast, circulating iron levels, which were assessed by measuring serum iron levels and transferrin saturation, decreased during the first 6 h following α-GalCer treatment and was followed by a significant increase between 24 and 48 h, with levels returning to normal by day 3 (Fig. 1C). In turn, hepcidin expression levels (*Hamp*) were elevated two-fold at 6 h after α-GalCer treatment, and then suppressed from 12 h and up to day 3 (Fig. 1D). Ferroportin 1 (*Fp1*) levels were rapidly suppressed starting at 3 h and up to 48 h after treatment (Fig. 1D). Overall, these data show that activation of iNKT cells by α-GalCer leads to biphasic changes in iron metabolism.

### Changes in iron metabolism induced by α-GalCer depend on the presence of CD1d and iNKT cells

**Figure 2:**
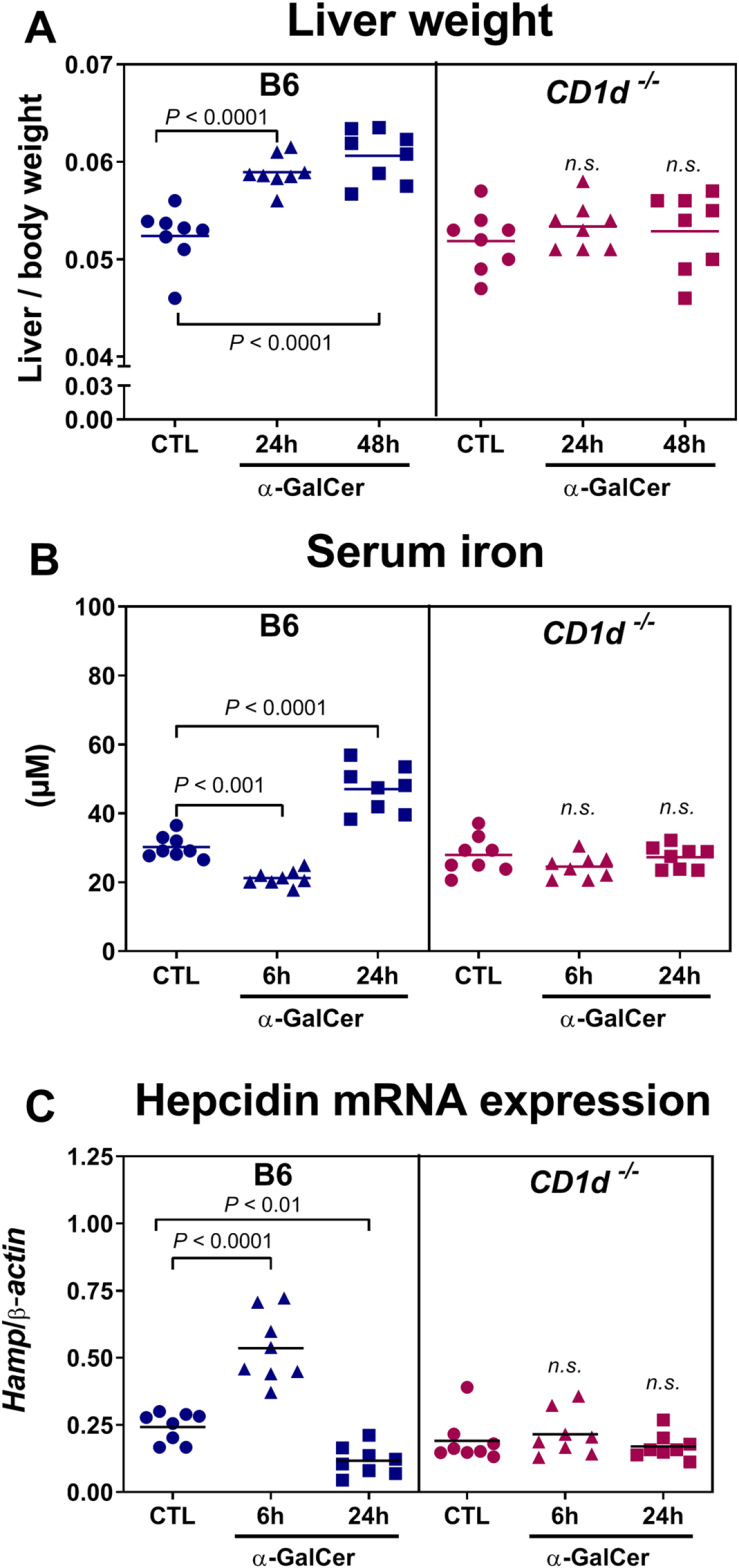
Iron metabolism changes induced by α-GalCer-mediated iNKT activation are abolished in *CD1d*^−/−^ mice. Wild-type and *CD1d*^−/−^mice were injected with vehicle (CTL) or 100 μg/Kg body weight of α-GalCer. (**A**) Liver weight. (**B**) Serum iron. (**C**) Hepcidin mRNA expression. Each symbol represents one mouse with the bar indicating the mean; *n* = 8 per group. Statistical analysis was performed with one-way ANOVA. *n.s.,* not significant compared to control mice (CTL) injected with vehicle.

α-GalCer loaded onto CD1d specifically binds to and activates iNKT cells. Therefore, the changes in iron metabolism following in vivo α-GalCer treatment most likely resulted from iNKT cell activation. To determine if the changes in iron metabolism were a direct or indirect consequence of iNKT cell activation by α-GalCer presented by CD1d, we used CD1d-deficient mice (*CD1d*^−/−^), which lack both iNKT and type 2 NKT cells^20^. After α-GalCer administration, *CD1d*^−*/*−^ mice had no variations in liver weight, and serum iron and hepcidin levels in the liver remained unaffected at 6 h and at 24 h (Fig. 2). These results demonstrate that α-GalCer treatment induces changes in iron metabolism in a CD1d-dependent manner.

**Figure 3:**
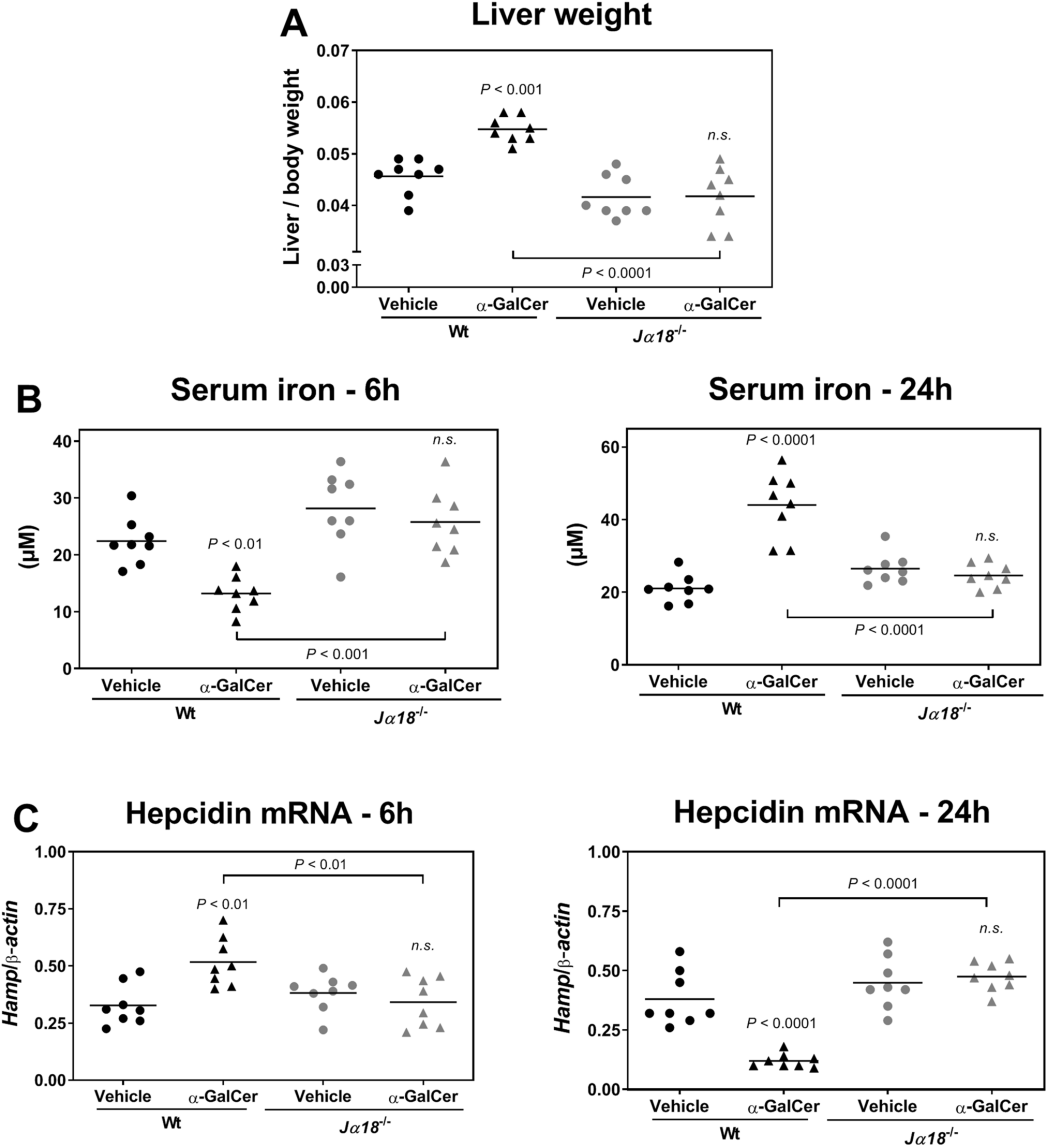
*Jα18*^−/−^ mice lacking Vα14 iNKT cells fail to respond to α-GalCer treatment and show no change in iron metabolism. Wild-type (Wt) and *Jα18*^−/−^ mice were injected with vehicle or 100 μg/kg body weight of α-GalCer. (**A**) Liver weight 24 h post-treatment. (**B**) Serum iron at 6 h and 24 h post-treatment. (**C**) Hepcidin mRNA expression at 6 h and 24 h post-treatment. Each symbol represents one mouse with the bar indicating the mean; *n* = 8 per group. Statistical analysis was performed with one-way ANOVA. *n.s. *not significant compared to control mice injected with vehicle.

Next, to determine if the absence of changes in iron metabolism following α-GalCer treatment in *CD1d*^−*/*−^ mice was either due to the absence of α-GalCer presentation by CD1d or due to the absence of NKT cells, we assessed the effect of α-GalCer on iron homeostasis using *Jα18*^−*/*−^ mice. These mice are deficient for the Jα18 TCR segment and specifically lack Vα14 iNKT cells, whereas all the other lymphoid cell lineages, including type 2 NKT cells, are intact^21^. The mice also express CD1d and are thus able to present α-GalCer. When compared to wild-type mice, liver weights in *Jα18*^−/−^ mice remained unchanged at 24 h post α-GalCer-treatment (Fig. 3A). Moreover, serum iron (Fig. 3B) and hepcidin mRNA expression (Fig. 3C) remained unaltered at both 6 h and 24 h following α-GalCer treatment. These results confirm that iNKT cells are indispensable for α-GalCer-induced changes in iron homeostasis and demonstrate that iNKT cell activation is sufficient to trigger prominent changes in iron homeostasis.

### The impact of α-GalCer on iron homeostasis is independent of NK cells

**Figure 4:**
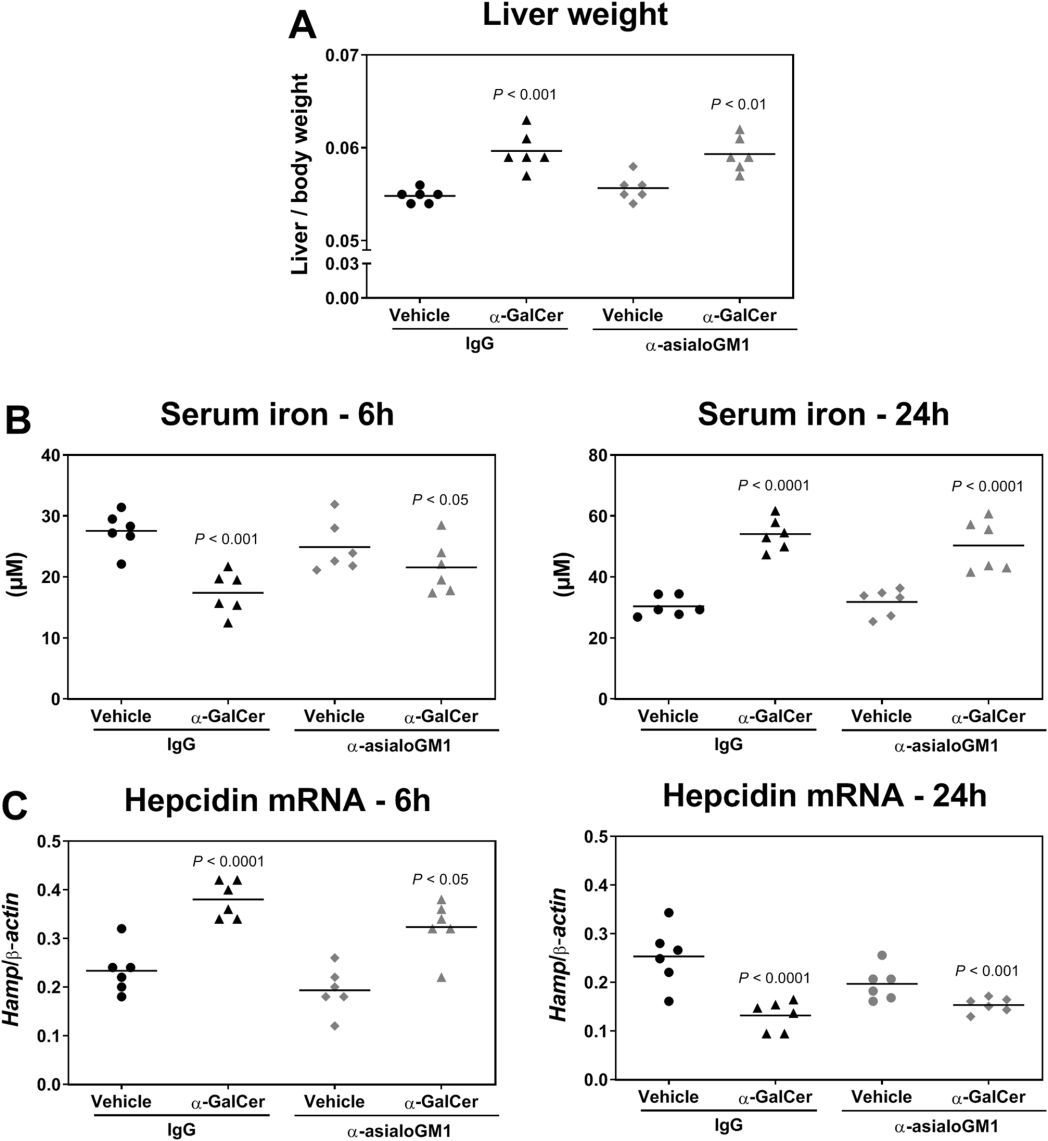
Iron metabolism changes induced by α-GalCer-mediated iNKT activation is independent of NK cells. Mice were injected with control isotype Ig (IgG) or with α-asialoGM1 antibodies before the administration of vehicle or 100 μg/Kg body weight of α-GalCer. (**A**) Liver weight 24 h post-treatment. (**B**) Serum iron at 6 h and 24 h post- α-GalCer treatment. (**C**) Hepcidin mRNA expression at 6 h and 24 h post- α-GalCer treatment. Each symbol represents one mouse with the bar indicating the mean; *n* = 6 per group. Statistical analysis was performed with one-way ANOVA.

We demonstrated that iNKT cell activation with α-GalCer affects iron homeostasis. We then investigated other cellular downstream effectors that may contribute to the changes in iron homeostasis. Considering that changes in iron metabolism occurred within hours of α-GalCer administration and that the changes did not persist beyond three days (Fig. 1), we examined the cellular components of innate immunity, such as NK cells, known to respond within this timeframe^15,22^. We depleted NK cells using the anti-asialoGM1 (α-AGM1) antibody^23^, which resulted in more than 75% depletion of NK cells in the liver (Supplementary Fig. 1). Two groups of mice, control isotype Ig-injected and α-AGM1 antibody-treated, were injected with α-GalCer or vehicle 24 h after NK cell depletion. As shown in Fig. 4, NK cell-depleted mice had similar responses to α-GalCer administration as control mice, showing an increase in liver weight at 24 h (Fig. 4A) as well as the biphasic changes in serum iron (Fig. 4B) and hepcidin mRNA expression (Fig. 4C). Indeed, while serum iron levels in both control and NK cell-depleted mice were reduced at 6 h and increased at 24 h, the hepcidin mRNA levels were conversely increased at 6 h and decreased at 24 h after α-GalCer treatment. Taken together, these results indicate that NK cells are not required for α-GalCer-induced disturbances in iron metabolism.

### Role of Kupffer cells in α-GalCer-induced changes in iron metabolism

**Figure 5:**
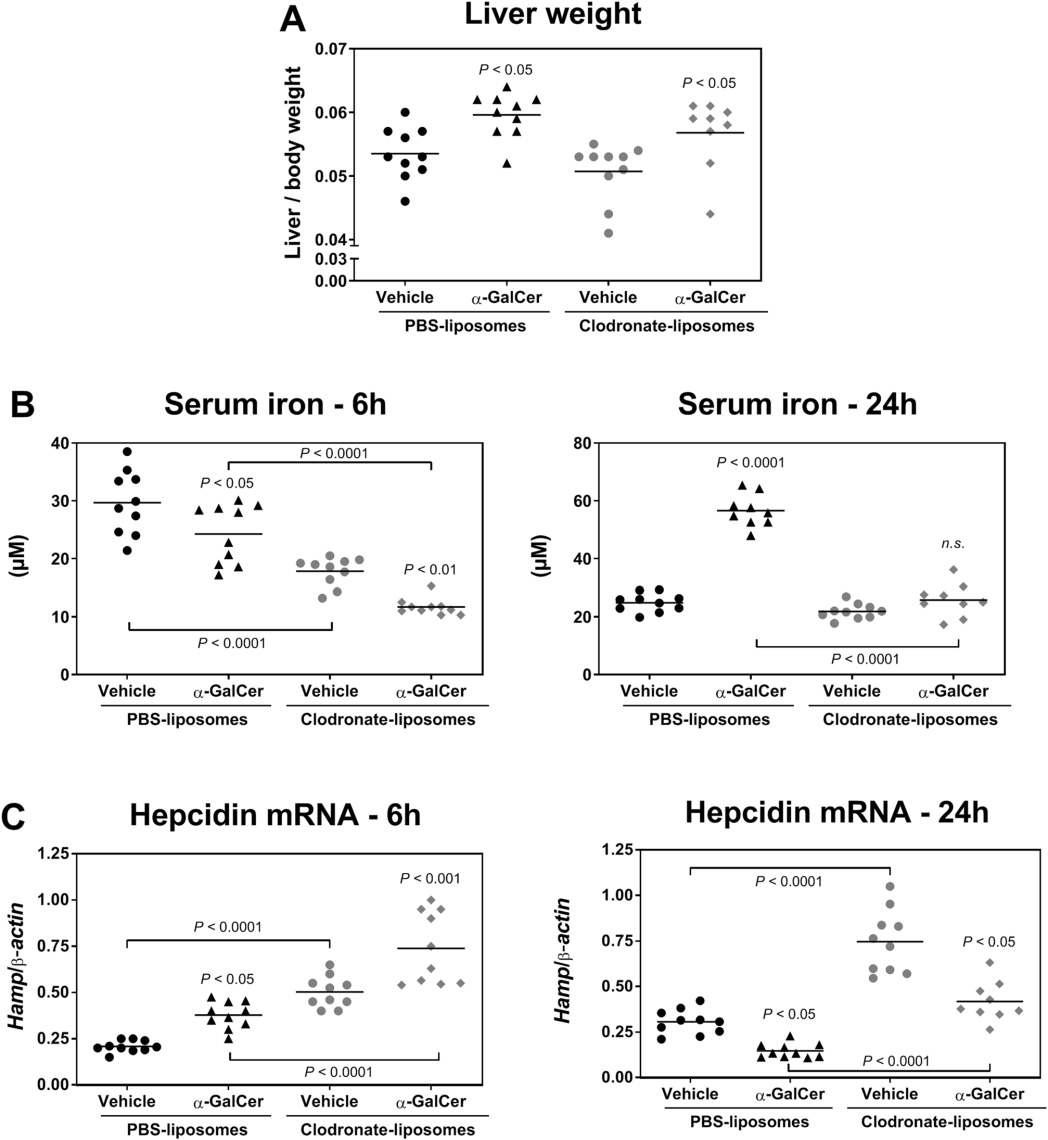
Influence of Kupffer cells on the iron metabolism changes induced by α-GalCer-mediated iNKT activation. Mice were injected with PBS-liposomes or with clodronate-liposomes 48 h before the administration of vehicle or 100 μg/Kg body weight of α-GalCer. (**A**) Liver weight 24 h post-treatment. (**B**) Serum iron at 6 h and 24 h post- α-GalCer treatment. (**C**) Hepcidin mRNA expression at 6 h and 24 h post-α-GalCer treatment. Each symbol represents one mouse with the bar indicating the mean; *n* = 9–10 per group. Statistical analysis was performed with one-way ANOVA. *n.s.* not significant compared to control mice injected with vehicle.

In addition to NK cells, Kupffer cells, the resident macrophages of the liver, are another likely cellular candidate that may contribute to α-GalCer-induced changes in iron metabolism as they produce vast amounts of IL-6^24^. Since IL-6 plays a major role in hepcidin regulation during inflammation^25^, we depleted Kupffer cells by pre-treating mice with clodronate liposome (c-lip) and assessed the depletion efficiency by RT-PCR using primers for Kupffer cell markers, F4/80 and Clec4f. When compared to control mice treated with phosphate-buffered saline-liposomes (PBS-lip), both F4/80 and Clec4f mRNA expression were significantly reduced in c-lip-treated mice, demonstrating that c-lip induced an efficient deletion of Kupffer cells in the liver (Supplementary Fig. 2). Importantly, the proportion and activation state of iNKT cells, as quantified by CD69 expression, was not influenced by c-lip treatment (Supplementary Fig. 3). PBS-lip-treated and c-lip-treated mice were subsequently challenged with α-GalCer treatment. Interestingly, the liver weights in both treatment groups were comparable and increased at 24 h post-α-GalCer treatment (Fig. 5A). This result suggests that depletion of Kupffer cells is not sufficient to prevent liver enlargement driven by iNKT cell activation.

We further examined the impact of Kupffer cell depletion on iron metabolism. When compared to PBS-lip-treated mice, c-lip-treatment alone (without α-GalCer treatment) resulted in significantly lower serum iron levels at 6 h but not at 24 h (Fig. 5B, comparison of vehicle treatment in PBS-lip and c-lip groups). This suggests that depletion of Kupffer cells per se has a transient impact on serum iron levels. Regardless, at 6 h following α-GalCer administration, serum iron levels decreased to a similar extent in both PBS-lip and c-lip treated mice (Fig. 5B), suggesting that Kupffer cells do not significantly contribute to the early phase of iron homeostasis disruption. At 24 h after α-GalCer-treatment, serum iron levels returned to normal levels in Kupffer cell-depleted mice injected with vehicle. However, α-GalCer treatment did not further elevate serum iron levels in Kupffer cell-depleted (c-lip-treated) mice relative to PBS-lip-treated mice. Altogether, these results suggest that Kupffer cells contribute to the maintenance of serum iron levels in the absence of acute stimulation and to the increase in serum iron levels following iNKT cell activation.

The acute hypoferremic response is accompanied by a strong induction of hepatic hepcidin mRNA expression^25^. Accordingly, at 6 h after vehicle administration, hepcidin mRNA levels were increased in c-lip-treated mice relative to PBS-lip treated mice, further supporting an impact of Kupffer cell deletion on iron metabolism in the absence of inflammatory challenge (i.e., without α-GalCer treatment, Fig. 5C). At 6 h post-α-GalCer treatment hepcidin mRNA levels were similarly increased in both PBS-lip- and c-lip-treated mice (Fig. 5C). Therefore, Kupffer cells do not contribute significantly to early changes in iron metabolism after α-GalCer treatment. In addition, hepcidin mRNA levels were similarly decreased at 24 h post-α-GalCer administration in both PBS-lip- and c-lip-treated mice (Fig. 5C). These results indicate that Kupffer cells are not essential for regulating hepcidin expression in response to α-GalCer treatment but contribute to hepcidin regulation under steady-state conditions.

### Liver damage after α-GalCer-mediated iNKT activation

**Figure 6:**
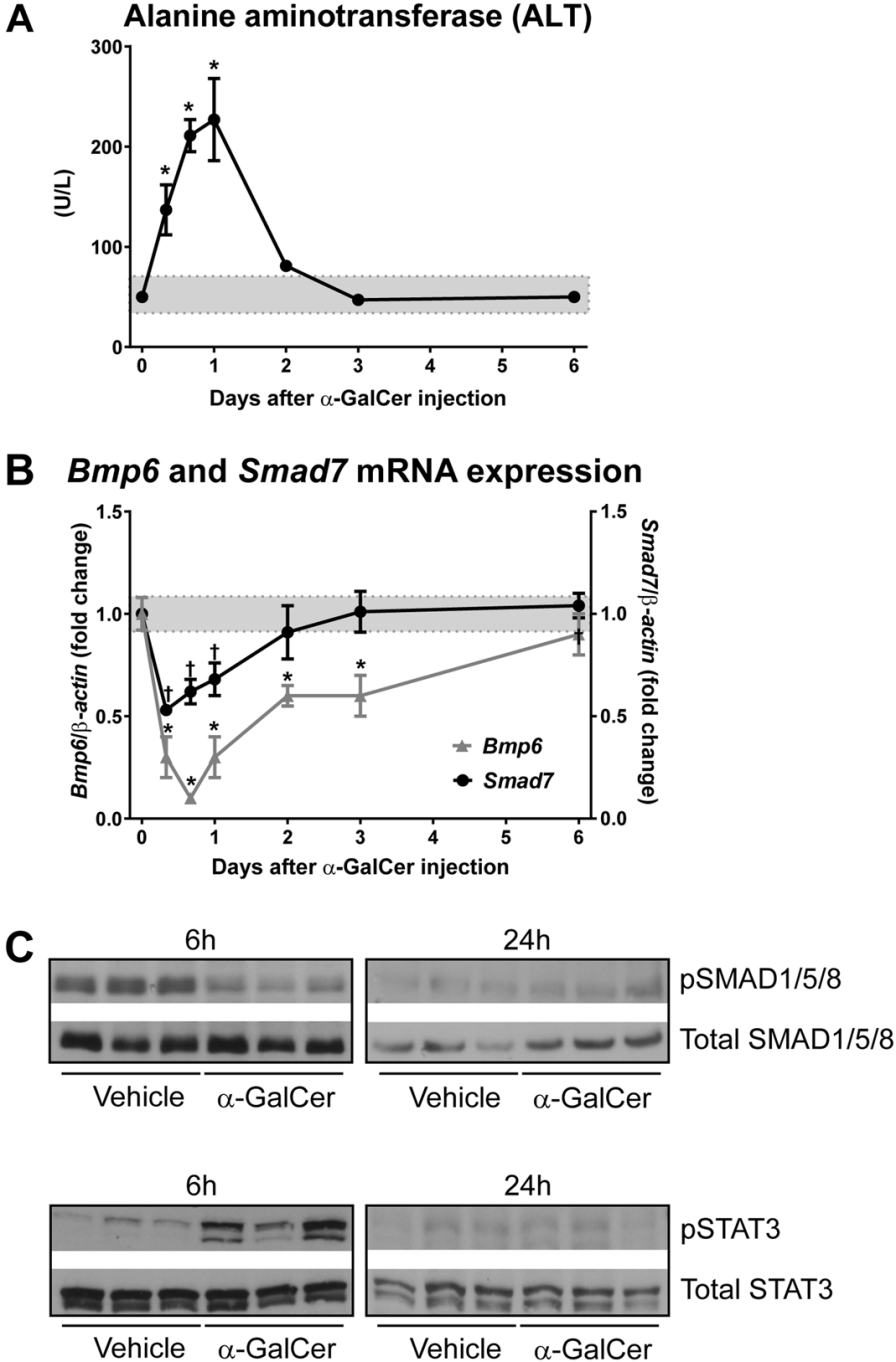
Liver damage induced by α-GalCer and inhibition of BMP/SMAD signaling pathway. Wild-type mice were injected with vehicle (indicated by the gray area across the graphs) or 100 μg/Kg body weight of α-GalCer. (**A**) Liver alanine aminotransferase levels. (**B**) *Bmp6* and *Smad7* mRNA expression in the liver. (**C**) Liver nuclear extracts analyzed by western blotting. Upper blots: phosphorylated SMAD1/5/8 (pSMAD1/5/8) and total SMAD1/5/8; Lower blots: phosphorylated STAT3 (pSTAT3) and total STAT3.The full-length blots are presented in Supplementary Fig. 6. Data in (**A**) and (**B**) are presented as mean ± SEM for a minimum of *n* = 12 mice per time point. Statistical analysis was performed with one-way ANOVA. *^,†^*P* < 0.01, compared to mice injected with vehicle at each time point.

Our results so far indicate that biphasic changes in iron homeostasis induced by α-GalCer are mediated by iNKT cells. The iNKT cell-driven impact on iron homeostasis does not involve the activation of NK cells, whereas Kupffer cells contribute to the regulation of serum iron levels and hepcidin expression in the absence of iNKT stimulation. In addition to inflammatory cytokine production, iNKT cells activated with α-GalCer can induce liver damage^26^, which could explain partially the increase in circulating and tissue iron levels. To quantify liver damage, we measured serum alanine aminotransferase (ALT) levels, which were elevated up to 24 h post-treatment with α-GalCer (Fig. 6A).

### Regulation of BMP/SMAD and STAT3 pathways after α-GalCer-mediated iNKT activation

Regulation of hepcidin expression by iron involves signaling through the bone morphogenetic protein (BMP) and Sma- and Mad-related protein 4 (BMP/SMAD4) pathway that regulates hepcidin via the SMAD1, SMAD5, and SMAD8 proteins^27^. This iron-signaling pathway involves upregulation of BMPs, particularly BMP6^28^, in response to heightened iron levels^29,30^. Hence, we next measured the levels of *Bmp6* mRNA expression levels in response to α-GalCer acute treatment. Despite the rise in serum and tissue iron, *Bmp6* mRNA levels substantially decreased as early as 6 h post-α-GalCer treatment (Fig. 6B).

Once the BMP6/SMAD pathway is activated by iron, inhibitory SMADs, particularly SMAD7, are also upregulated^31^. However, *Smad7* mRNA levels were significantly suppressed after α-GalCer administration (Fig. 6B). This indicates that the lack of response to elevated iron levels was not due to an exaggerated expression of SMAD7, but that instead, *Smad7* expression levels appeared to follow a general pattern of inhibition of BMP/SMAD signaling in response to α-GalCer.

To further confirm that hepcidin suppression in response to α-GalCer treatment was due to inhibition of the BMP/SMAD pathway, we assessed phosphorylation levels of SMAD1/5/8 in liver nuclear extracts. We found that SMAD1/5/8 phosphorylation was decreased at 6 h and 24 h post-α-GalCer treatment (Fig. 6C), confirming that the BMP/SMAD pathway was not activated in response to elevations in circulating and tissue iron induced by α-GalCer.

Hepcidin levels are additionally regulated by inflammatory cytokines, such as IL-6, via the signal transducer and activator of transcription 3 (STAT3) pathway^32^. Accordingly, we found that at 6 h but not at 24 h post-treatment, STAT3 phosphorylation was enhanced, which was consistent with hepcidin induction during the early phase (Fig. 6C). Overall, these data indicate that at earlier stages after iNKT activation, STAT3 signaling is enhanced while BMP/SMAD signaling is suppressed. The outcome of these opposing signals of *Hamp* induction via STAT3 phosphorylation vs. *Hamp* inhibition via BMP/SMAD suppression, is the activation of *Hamp* expression via the inflammatory, STAT3-mediated pathway. In contrast, inhibition of the BMP/SMAD signaling pathway predominates at the stage when STAT3 activation ceases, and consequently *Hamp* expression is repressed.

### α-GalCer treatment induces immune cell proliferation in the liver and spleen

**Figure 7:**
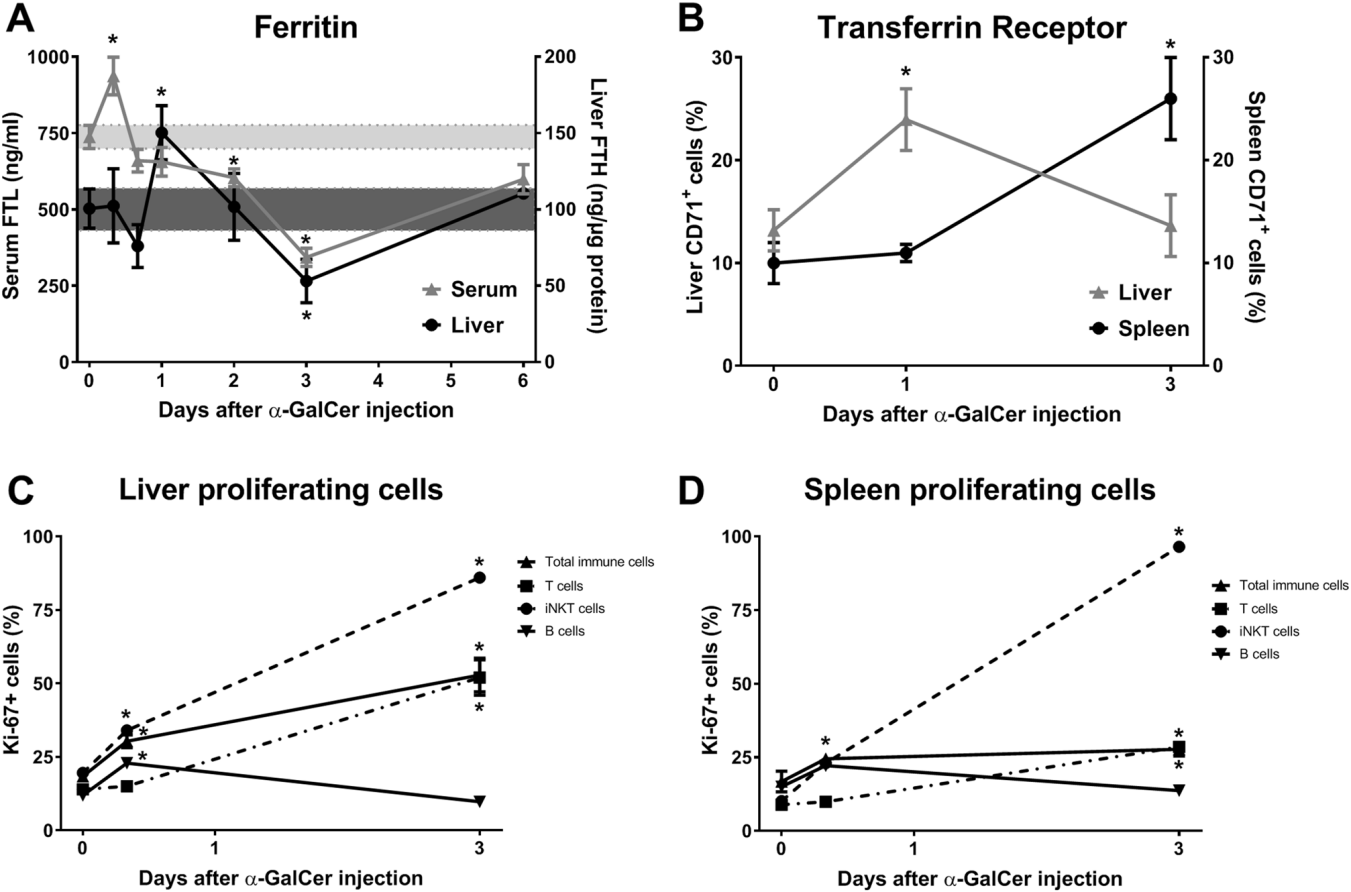
Serum and liver ferritin changes and immune cell proliferation induced by α-GalCer. Wild-type mice were injected with vehicle or 100 μg/Kg body weight of α-GalCer. (**A**) Serum and liver ferritin levels. (**B**) Transferrin receptor (CD71) expression within mononuclear cells isolated from liver and spleen. (**C**) Proliferating cells in the liver. (**D**) Proliferating cells in the spleen. Data are presented as mean ± SEM for a minimum of *n* = 3–6 mice per time point. Statistical analysis was performed with one-way ANOVA. *P < 0.05, compared to mice injected with vehicle.

To further understand the increase in total liver iron induced by α-GalCer treatment (Fig. 1B), we measured circulating and liver ferritin levels. Serum ferritin levels increased at 6 h, followed by a significant decrease starting at 48 h to day 3, while liver ferritin levels followed a similar kinetic by increasing with a slight delay at 24 h and significantly decreasing at 72 h (Fig. 7A). Ferric iron staining of liver samples by DAB-enhanced Perl’s Prussian blue showed a similar distribution pattern (Supplementary Fig. 4). These results indicate that the extra iron in the liver was not being stored in ferritin or hemosiderin. Since iron is required for DNA synthesis in proliferating cells^33^, where it would not be stored in ferritin, we directly assessed the levels of CD71 or transferrin receptor (TfR) on mononuclear cells isolated from the liver and spleen using flow cytometry. The percentage of cells expressing CD71 was significantly elevated at 24 h in the liver and at 72 h in the spleen (Fig. 7B). These data suggest that immune cells were increasing their ability to uptake iron for proliferation rather than for storage into ferritin.

α-GalCer treatment induces activation and proliferation of iNKT cells^14,15^. These activated iNKT cells produce cytokines, which lead to the recruitment, activation and proliferation of other immune cells^16,17^. Indeed, we found that at day 3, the proportion and absolute number of iNKT cells was significantly increased in the liver of α-GalCer-treated mice relative to controls (Supplementary Fig. 5). In line with this increase in number, we found that iNKT cells were actively proliferating, as quantified by the expression of Ki-67 (Fig. 7C,D). The activation of iNKT cells also resulted in the proliferation of other immune cells. Using CD45 to identify all immune cells, we noted an increase in the Ki-67 proliferation marker as early as 6 h following α-GalCer administration (Fig. 7C,D). Whereas B cell proliferation was modestly increased, T cell proliferation was significantly induced in both the liver and the spleen (Fig. 7C,D). Overall, α-GalCer-mediated activation of iNKT cells induced substantial immune cell proliferation in both the liver and spleen. Notably, the proliferation was more prominent in the liver, where iNKT cells are most abundant.

## Discussion

The aim of this study was to investigate the influence of activated iNKT cells on iron homeostasis. Our work suggests that iNKT activation has particular effects in iron homeostasis that could be relevant for treatments aimed at regulating hepcidin expression. A possible role for iNKT cells in modifying iron metabolism is suggested by their ability to secrete cytokines upon activation, and by their predominant location in mouse liver, where the major regulator of iron homeostasis, hepcidin, is produced. Hepcidin regulates systemic iron homeostasis via the hepcidin/ferroportin axis, as it binds to ferroportin 1 and induces ferroportin internalization and degradation^34^. Decreased ferroportin levels result in lowering intestinal iron absorption and iron export from macrophages and Kupffer cells, which are responsible for iron recycling. Therefore, hepcidin levels are tightly regulated by inflammation, anaemia, hypoxia, and iron levels^35^, and the mechanisms that are involved overlap to control immune responses, prevent iron toxicity, and respond to changes in erythropoietic demand^36^. Here, we show that α-GalCer-mediated iNKT cells activation in vivo results in biphasic changes in systemic iron homeostasis.

The changes in iron metabolism triggered by α-GalCer were abolished in *CD1d*^−/−^ mice. Since α-GalCer is a highly specific ligand for CD1d, this rules out possible unspecific effects, including those unrelated to antigen presentation. This also highlights a specific role for iNKT cells, confirmed by the lack of response in *Jα18*^−/−^ mice. Indeed, while *CD1d*^−/−^ mice lack all NKT cell subsets, *Jα18*^−/−^ mice are specifically devoid of Vα14 iNKT cells^21^. Interestingly, iron in turn may influence iNKT levels, as shown in patients with hereditary hemochromatosis, an iron overloading disease. These individuals show a decrease in iNKT cells that is more pronounced in patients with higher iron levels^37^. Finally, using anti-asialoGM1 antibodies, we ruled out a role for NK cells, which undergo proliferation and activation after α-GalCer administration.

Kupffer cells, the resident macrophages of the liver, have been historically studied in regard to iron metabolism as they are major producers of pro-inflammatory cytokines. In addition, Kupffer cells and macrophages play a fundamental role in iron recycling after erythrophagocytosis^38^. In our study, c-lip depletion of Kupffer cells alone significantly altered iron homeostasis, as shown by a decrease in serum iron accompanied by increased hepcidin expression. Similar effects after Kupffer cell depletion have been previously reported^39^. We demonstrate that these changes are not due to secondary activation of iNKT cells following Kupffer cell depletion. In addition, our depletion experiments demonstrated that Kupffer cells are dispensable for α-GalCer-induced modulation of hepcidin. Our findings support and add to previous work by others, which demonstrated that Kupffer cells do not play a role in regulating hepcidin expression in response to inflammation^39,40^ and iron^39–41^.

We followed iron-related parameters after a single injection of α-GalCer to activate iNKT cells in C57BL/6 wild-type mice. Upon injection of α-GalCer, an early response was observed with decreases in serum iron, transferrin saturation, and *Fp1* expression, while hepcidin expression increased. These early responses are similar to the changes observed upon lipopolysaccharide injection, which are attributed to inflammatory cytokines^32,42^ and are consistent with the well-documented ability of activated NKT cells to rapidly release copious amounts of the proinflammatory cytokines IFN-γ, TNF-α, and IL-6 in circulation and in the liver^43,44^. Accordingly, we observed an increase in STAT3 phosphorylation at 6 h post-treatment. This may explain the parallel induction of hepcidin at this early phase, as STAT3 activation has been shown to induce hepatic hepcidin expression in response to inflammation^32^. Interestingly, at the same 6 h time point, SMAD1/5/8 phosphorylation was downregulated, which would downregulate hepcidin expression towards hepcidin suppression. This agrees with previous work showing that the level of hepcidin expression levels in the presence of opposing signaling is determined by the strength of the individual stimuli which, in our study, is the upregulation of STAT3 phosphorylation^36^.

A second phase following α-GalCer treatment involved a rise in tissue and serum iron levels but, paradoxically, hepcidin levels were strongly inhibited. The increase in circulating and tissue iron levels may be partially due to liver damage, since administration of α-GalCer to mice has been shown to induce immune-mediated liver injury and cell death^26^ that may be accompanied by iron release from dying cells. Our results corroborate these earlier studies, based on our observation of elevated serum ALT levels that peaked between 12 and 24 h post-treatment. Alternatively, or concomitantly, Kupffer cell may also be involved in the increase in serum iron levels, since their depletion seemed to abolish the serum iron surge at 24 h.

The rise in iron levels would be expected to modulate hepcidin levels by inducing its expression, as found in mice treated with iron-enriched diets^6,45,46^ or injected with iron-dextran^6^. At 24 h after acute α-GalCer treatment, STAT3 phosphorylation returned to normal. Hence, non-inflammatory pathways regulate hepcidin expression at the later stage/second phase. The iron-sensing pathway initiates a sequence that starts with an increase in BMP levels, notably BMP6^30,47^, binding to BMP receptors, followed by activation of SMAD1/5/8/phosphorylation^47^ and binding to SMAD4^48^. The resulting SMAD complex translocates into the nucleus and binds to the BMP responsive element in the *Hamp* promoter region^49^.

Previous studies demonstrated that both circulating and tissue iron activate the BMP/SMAD signaling pathway to modulate hepcidin expression, albeit targeting different levels of the pathway^29^. While elevation of liver iron levels induces hepatic expression of *Bmp6* in the liver, increased transferrin saturation activates SMAD1/5/8 phosphorylation downstream of BMP6. Intriguingly, in mice treated with α-GalCer, liver *Bmp6* levels were strongly suppressed, and SMAD1/5/8/phosphorylation did not increase despite elevated levels of circulating iron. This suggests that the inhibition of hepcidin mRNA expression is related to a lack of activation of the BMP6/SMAD pathway in response to elevated circulating iron. In our setting, *Smad7* expression was also significantly suppressed, ruling out a possible activation of inhibitory SMAD7, which functions as a feedback inhibitor of the BMP/SMAD pathway^31^. In addition, inhibitory SMAD7 was previously shown to follow the overall activation of the SMAD1/5/8 signaling pathway and thus, was significantly modulated by both acute and chronic iron administration^29^. Therefore, downregulation of *Smad7* expression in our studies further supports the view that α-GalCer suppressed hepcidin expression via inhibition of the BMP6/SMAD pathway.

A decrease of hepatic *Bmp6* mRNA levels in the presence of heightened liver iron has also been described in conditional knockout models of the iron exporter ferroportin^50^. These mouse models showed decreased *Bmp6* expression and phosphorylated SMAD1/5/8 as well as decreased expression of *Hamp* in the liver, despite elevated liver iron loads. The investigators attributed the decrease in *Bmp6* mRNA to a liver-independent signal generated in response to high iron demand for erythropoiesis. In our study, we show that α-GalCer strongly stimulates the proliferation of immune cells, including NKT, B and T lymphocytes, as shown by others^18,19,26^. Moreover, lymphocytes are known to increase the synthesis and expression of surface TfR in response to the increased iron demand during proliferation^51^. Accordingly, we also show a significant increase in cells expressing TfR, consistent with the need to increase transferrin uptake to provide iron for fundamental cellular processes, especially for DNA synthesis^33^. Heightened iron demand to support immune cell proliferation in response to α-GalCer may partially explain the inhibition of the BMP/SMAD pathway and, consequently, the downregulation of *Hamp* expression.

In summary, the present work describes the effects of α-GalCer on iron homeostasis and demonstrates that iNKT cells are essential for α-GalCer effects. Activation of iNKT cells in vivo triggers substantial immune cell proliferation that translated into increased iron demand, contributing to a disassociation between temporary rises in circulating and tissue iron, and a marked suppression of hepcidin expression in the liver via inhibition of the BMP/SMAD pathway. Altogether, our findings suggest that the iNKT/CD1d system plays an important role in iron homeostasis and that modulation of the iNKT/CD1d pathway may be useful to regulate hepcidin expression.

## Methods

### Mice

This study was carried out in accordance with Canadian Council on Animal Care guidelines. The protocol was evaluated and approved by the institutional Animal Care Committee of the CRCHUM (“Supplementary methods”). C57BL/6 wild-type (Wt), and *CD1d*^−/−^ (C57BL/6 background) mice, were purchased from Jackson Laboratories (Bar Harbor, ME). Mice deficient in iNKT cells (*Jα18*^−/−^ mice) were kindly provided by Dr. Taniguchi (Kanagawa, Japan)^21^ and were backcrossed at least 10 times to C57BL/6 (Dr. Thierry Mallevaey, University of Toronto, Canada)^52^.

### Measurement of serum iron, transferrin saturation, ALT, and tissue iron concentration

Serum iron, total iron‐binding capacity, transferrin saturation, and ALT were assessed via colorimetric assay with the Kodak Ektachem DT60 system (Johnson & Johnson, Ortho Clinical Diagnostics, Mississauga, ON). Spleen and liver iron concentrations were assessed by acid digestion of tissue samples, followed by iron quantification with atomic absorption spectroscopy^53^.

### In vivo activation of iNKT cells

Mice received one injection of 100 μg/kg body weight of α-GalCer (Enzo Life Sciences Inc., Farmingdale, NY) diluted in Dulbecco's modified eagle medium (DMEM) (Wisent Inc., St-Bruno, Québec, Canada). Control mice received a corresponding dose of vehicle.

### NK and Kupffer cell depletion

NK cells were depleted by intraperitoneal injection of 500 μg anti-asialo GM1 antibody (α-AGM1; Wako Chemicals, Richmond, VA, USA) diluted in PBS, 24 h before α-GalCer treatment. Control mice were injected on the same days with 500 μg control rabbit Ig (Jackson ImmunoResearch, West Grove, PA, USA). To deplete Kupffer cells, 200 μL of clodronate liposome suspension (c-lip; 5 mg/ml; ClodronateLiposomes.org, Haarlem, The Netherlands) or of PBS encapsulated liposomes (PBS-lip) as control, was injected intravenously into the mice 48 h before administration of α-GalCer^54,55^.

### Confirmation of NK and Kupffer cells depletion

Depletion of NK cells was confirmed by analyses of liver mononuclear cells stained with anti-NK1.1 phycoerythrin-conjugated monoclonal antibody (mAb) and anti-CD3 Alexa fluor-conjugated mAb (both from PharMingen, San Diego, CA), followed by flow cytometry as previously described^45^. Samples were analyzed using an LSRFortessa flow cytometer (BD Biosciences) and the data were analyzed using FlowJo software (BD Biosciences).

Kupffer cell depletion was confirmed by quantitative RT-PCR in liver samples using primers for HPRT-1, F4/80 as a representative surface marker of mouse mononuclear phagocytes^56^, and Clec4f, a specific marker of Kupffer cells^57^.

### Measuring iNKT cell proportion and activation following c-lip treatment

Livers were perfused with cold PBS followed by a solution of 1 mg/ml of collagenase type V (Sigma). Livers were then harvested, cut into small pieces and incubated with 5 ml of collagenase type V for 20 min at 37 °C. The enzymatic digestion was stopped with ice cold media containing 10% fetal calf serum. The liver was pressed through a 70 µm cell strainer. The cell suspension was washed, and lymphocytes were separated on a 33.3% Percoll density gradient. Spleens were harvested and pressed through a 70 µm cell strainer. Both cell suspensions from liver and spleen were treated with an NH_4_Cl solution to lyse red blood cells. Single cell suspensions of liver and spleen were counted on a hemacytometer using trypan blue. To quantify iNKT cells and determine their activation status, cells were stained with the following reagents from Biolegend unless otherwise specified: Viability dye (Zombie Aqua™), CD45 PerCP-Cy5.5 (RA3-6B2), CD3 BV711 (17A2), CD19 Alexa-Fluor 700 (6D5), PBS57-coupled CD1d tetramer Pacific Blue (NIH tetramer core facility), CD69 APC (H1.2F3), CD335 PE (29A1.4, eBioscience), and NK1.1 BV605 (PK136). The samples were acquired on BD FACSCelesta and analysed with FlowJo LLC software version 10 (BD Biosciences). iNKT cells were defined as CD45^+^ CD19^−^ CD3^+^ CD1d-tetramer^+^ CD335^−^ NK1.1^+^ cells. The percentage of CD69^+^ cells among iNKT cells reflects the activation status. Dead cells and doublets were excluded from the analyses.

### Measuring immune cell proportion and proliferation following αGal-Cer treatment

At the indicated time points, single cell suspensions of livers and spleens were prepared as described above. To quantify cell proliferation, cells were stained with the same combination of antibodies used to identify iNKT cells in addition to Ki-67 FITC (B56, BD Biosciences). As a measure of proliferation, the percentage of Ki-67^+^ cells was quantified on total hematopoietic cells (CD45+), T cells (CD45^+^ CD3^+^ CD19^−^), B cells (CD45^+^CD3^−^CD19^+^) and iNKT cells (CD45^+^ CD19^−^ CD3^+^ CD1d-tetramer^+^ NK1.1^+^). To quantify TfR expression, cells were stained with the CD71 FITC antibody (C2, BD Biosciences). The samples were acquired on BD FACSCelesta and analysed with FlowJo LLC software version 10 (BD Biosciences). Dead cells and doublets were excluded from the analyses.

### Quantitative RT-PCR

Total RNA was isolated with Trizol reagent (Invitrogen, Burlington, ON), and reverse transcription was performed with the Omniscript RT kit (QIAGEN, Mississauga, ON). mRNA expression levels were measured by real-time PCR in a Rotor Gene 3000 Real Time DNA Detection System (Montreal Biotech, Kirkland, QC) with QuantiTect SYBRGreen I PCR kits (QIAGEN, Mississauga, ON) as previously described^53^. Expression levels were normalized to the housekeeping gene β*-*actin (*Actb*). Primers are listed in Supplementary Table 1.

### SDS–PAGE and Western blot analysis

SDS–PAGE and western blot analysis were performed on liver nuclear extracts^36^. Livers were removed, rinsed in ice-cold PBS, and used to prepare liver nuclear extracts with Nuclear Extract Kits (Active Motif, Carlsbad, CA). Nuclear protein extracts were separated by 10% SDS–PAGE gel and blotted onto nitrocellulose membranes (GE Healthcare, Little Chalfont, United Kingdom). The membranes were immunoblotted with the following antibodies: phospho-STAT3, STAT3, phospho-SMAD1/5/8 (Cell Signaling, catalogue #9511S, Danvers, MA), SMAD1/5/8 (Santa Cruz Biotechnology, catalogue #SC-6031-R, Santa Cruz, CA) and β-actin (Abcam, Cambridge, MA). As a secondary antibody, anti–rabbit IgG (Cell Signaling) or anti–mouse IgG (GE Healthcare) were used. Antigen–antibody complexes were visualized with the ECL Western Blotting Detection Reagent (GE Healthcare) in x-ray film. Films were digitalized using a scanner (HP psc2350, 300 dpi resolution), and obtained JPEG images were imported into Adobe Illustrator (300 dpi) and assembled.

### Serum and liver ferritin assay

Ferritin was measured with an ELISA kit as per manufacturer's instructions for serum (mouse Ferritin Light chain ELISA kit, Kamiya Biomedical, Seattle, USA) and for liver homogenates (mouse Ferritin Heavy chain ELISA kit, Kamiya Biomedical).

### Histology

Liver tissue sections were stained with DAB-enhanced Perl’s Prussian blue for ferric iron detection (iron stain kit; Sigma Immunochemicals).

### Statistical analysis

All statistics were calculated with Prism software (GraphPad, San Diego, CA), with a pre-specified significant *P*-value of 0.05. Data were pooled from two or three independent experiments. Multiple comparisons were evaluated statistically by one-way analysis of variance (ANOVA) followed by the Bonferroni multiple comparison test.

## Supplementary information

Supplementary Information.
