# Supplementary material for: Acute invariant NKT cell activation triggers an immune response that drives prominent changes in iron homeostasis
Source: Sci Rep. 2020 Dec 3;10:21026. doi: 10.1038/s41598-020-78037-3 (PMC7713400; doi:10.1038/s41598-020-78037-3)
Supplement: Supplementary file 1 — Supplementary Information. [file 41598_2020_78037_MOESM1_ESM.docx]

**Supplementary information**

**Acute invariant NKT cell activation triggers an immune response that drives prominent changes in iron homeostasis**

Hua Huang, Vanessa Zuzarte Luis, Gabriela Fragoso, Annie Calvé, Tuan Anh Hoang, Manon Oliero, Geneviève Chabot-Roy, Victor Mullins-Dansereau, Sylvie Lesage, Manuela M. Santos

**Supplementary Figures**


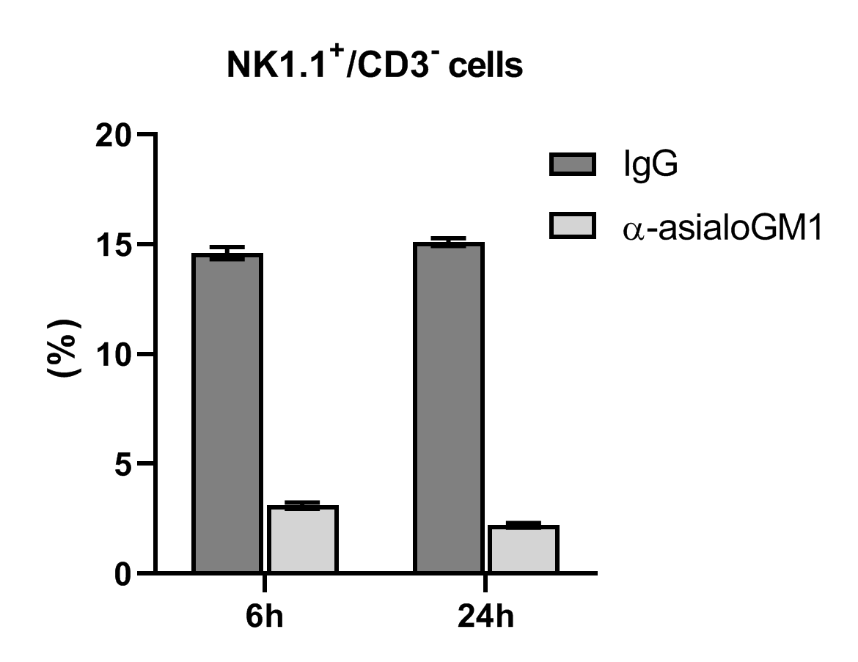


**Supplementary Figure 1**

**NK depletion with anti-asialoGM1 antibodies.** Liver mononuclear cells were isolated, and the depletion efficiency was verified by flow cytometry. The percentage of NK1.1-positive/CD3-negative (NK.1.1^+^/CD3^-^) cells among liver mononuclear cells isolated from livers are shown at 6 h and 24 h post-α-GalCer treatment. Data are presented as mean ± SEM.


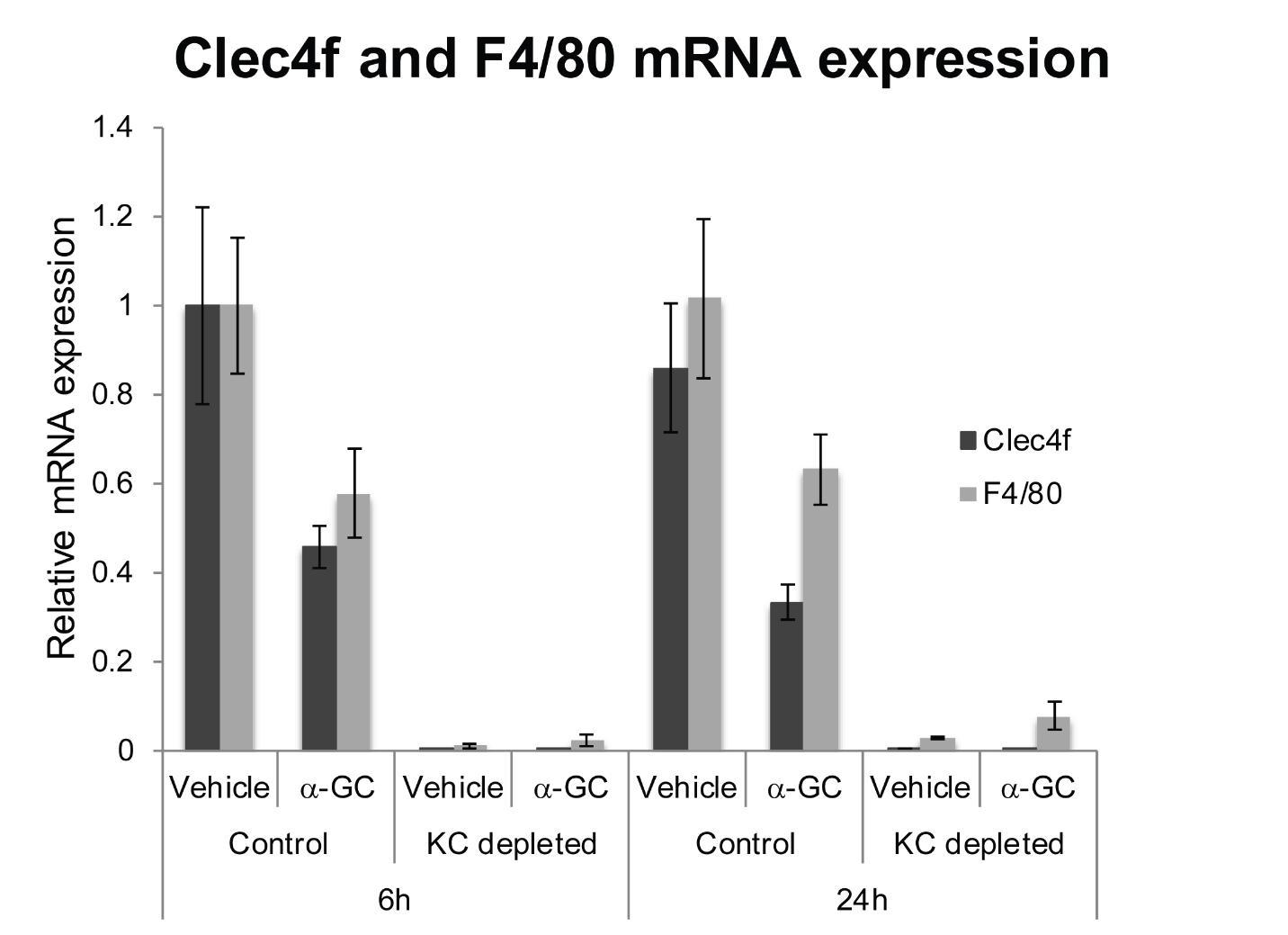


**Supplementary Figure 2**

**Kupffer cell depletion using clodronate liposomes.** Total RNA was isolated from liver samples. Reverse transcription was performed, and mRNA expression levels were measured by quantitative PCR. Hepatic mRNA expression of Clec4f and F4/80 in mice treated with PBS-liposomes (control) or clodronate liposomes (KC depleted) at 6 h and 24 h after vehicle or α-GalCer (α-GC) treatment. Data are presented as mean ± SD.


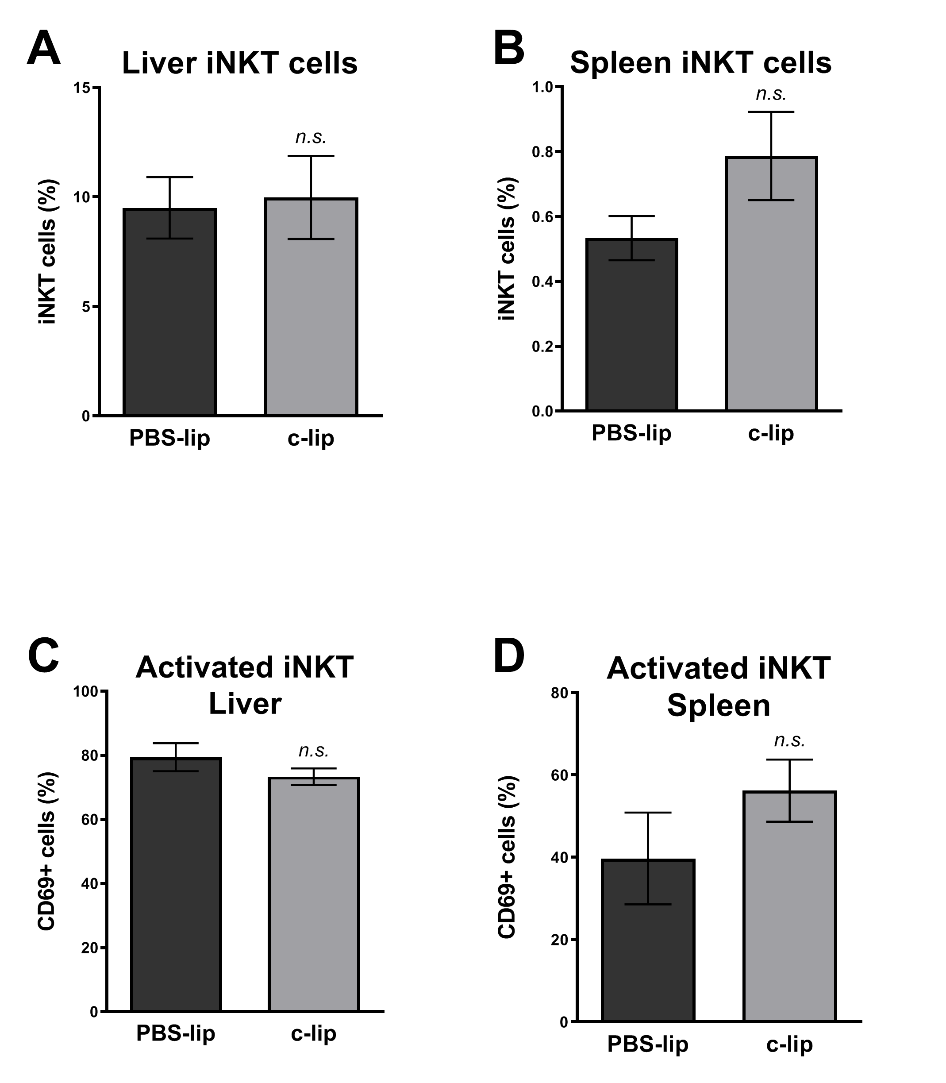


**Supplementary Figure 3**

**Kupffer cell depletion has no impact on iNKT cells.** Mice were injected with PBS-liposomes (PBS-lip) or clodronate liposomes (c-lip) two days prior to the isolation of immune cells. Kupffer cell depletion in the liver and myeloid cell depletion in the spleen was confirmed by flow cytometry (not shown). The percentage of iNKT cells was quantified in the **(A)** liver and **(B)** spleen based on the expression of CD3^+^CD1d-tetramer^+^NK1.1^+^ cells among CD45^+^ hematopoietic cells. The percentage of activated iNKT cells expressing CD69 in **(C)** liver and **(D)** spleen is shown. Data are presented as mean ± SD. *n.s.,* not significant compared to control mice injected with PBS-lip, Mann-Whitney U test.

**
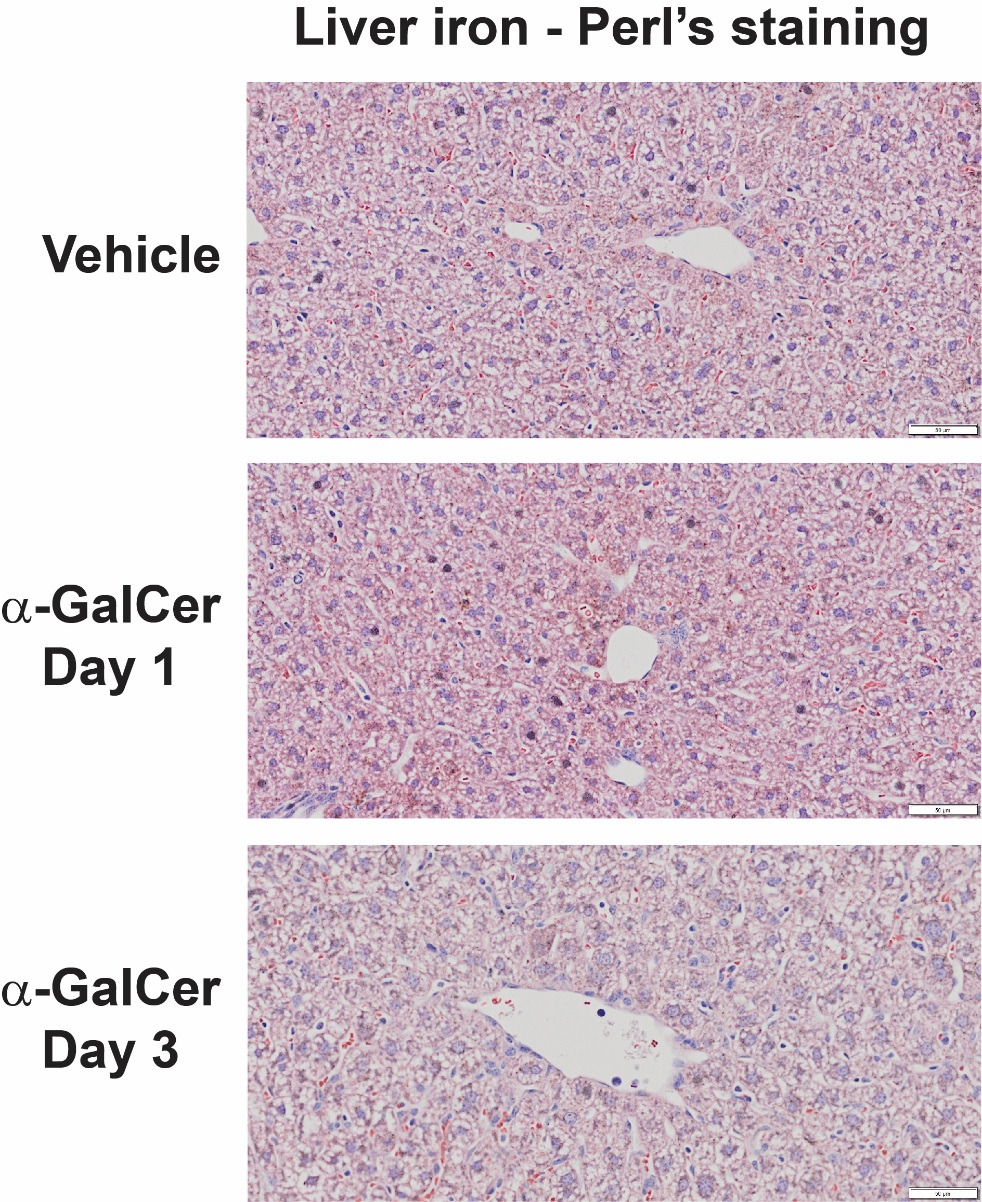
**

**Supplementary Figure 4**

**Ferric iron staining in liver sections.** Iron levels in the liver of mice 24 h and 72 h after treatment with vehicle or with α-GalCer, detected by DAB-enhanced Perl's staining (brown) with H&E counterstaining.

**
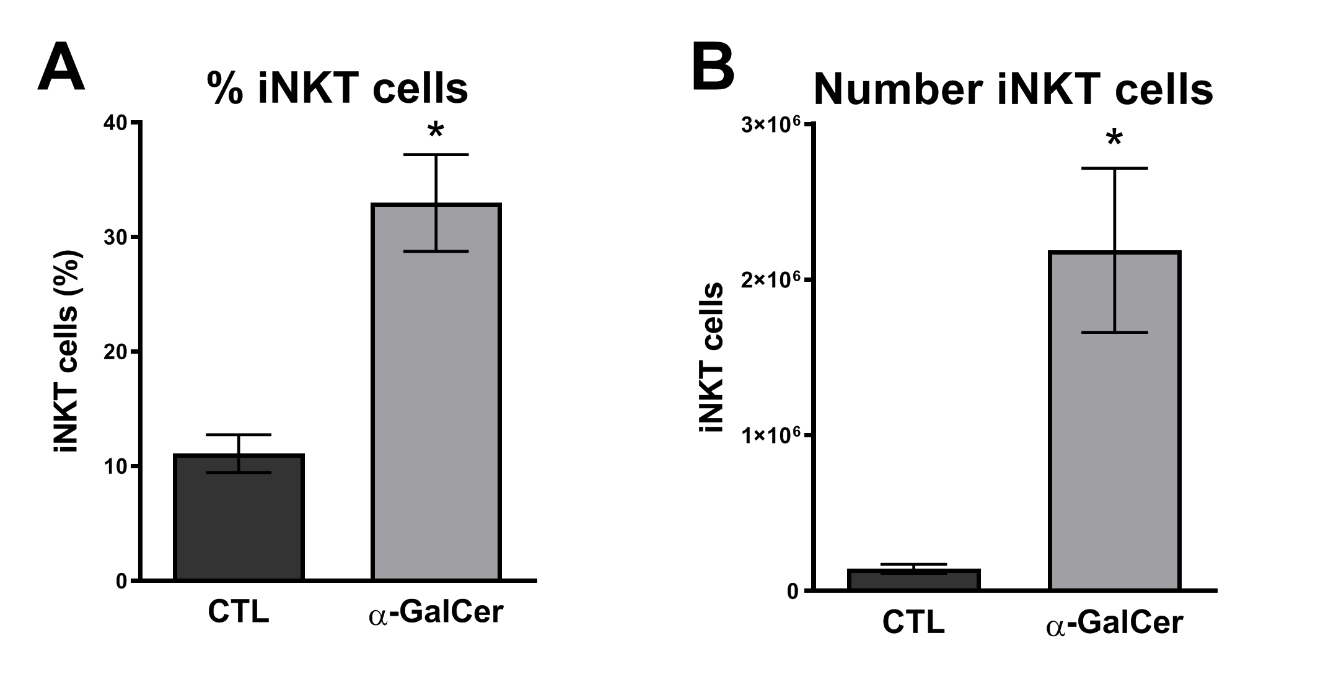
**

**Supplementary Figure 5**

**Treatment with α-GalCer increases the number of iNKT cells**. Mice were untreated (CTL, control) or injected with α-GalCer three days prior to the isolation of immune cells. **(A)** Percentage and **(B)** absolute number of iNKT cells in the liver, as quantified based on the expression of CD3^+^CD1d-tetramer^+^NK1.1^+^ cells among CD45^+^ hematopoietic cells. Data are presented as mean ± SEM. **P* < 0.05, Mann-Whitney U test.

**Supplementary Figure 6**

Original, unprocessed versions of the western blots shown in Figure 6C.

**
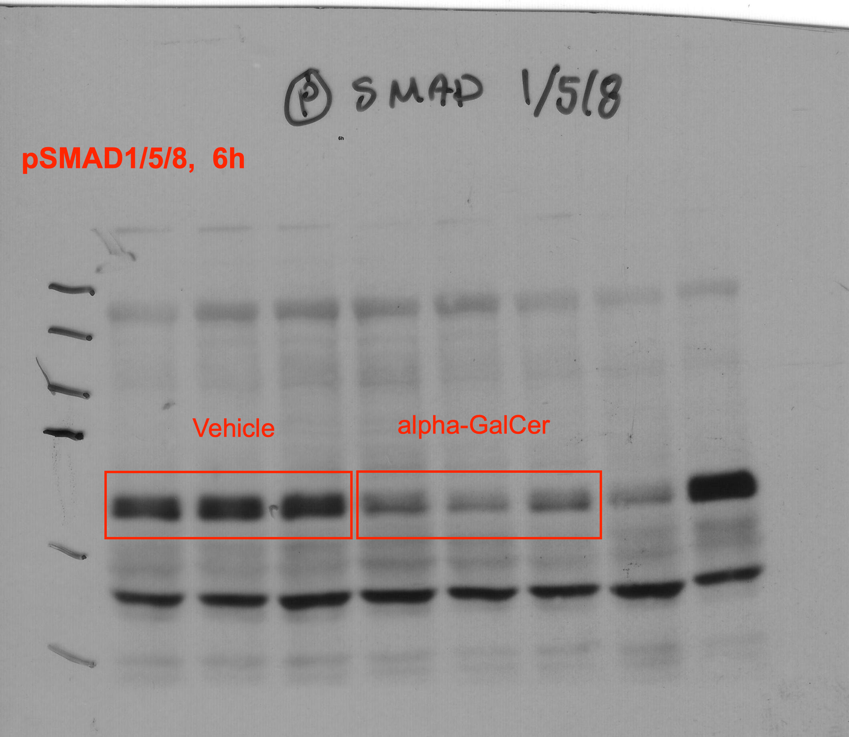
**

**
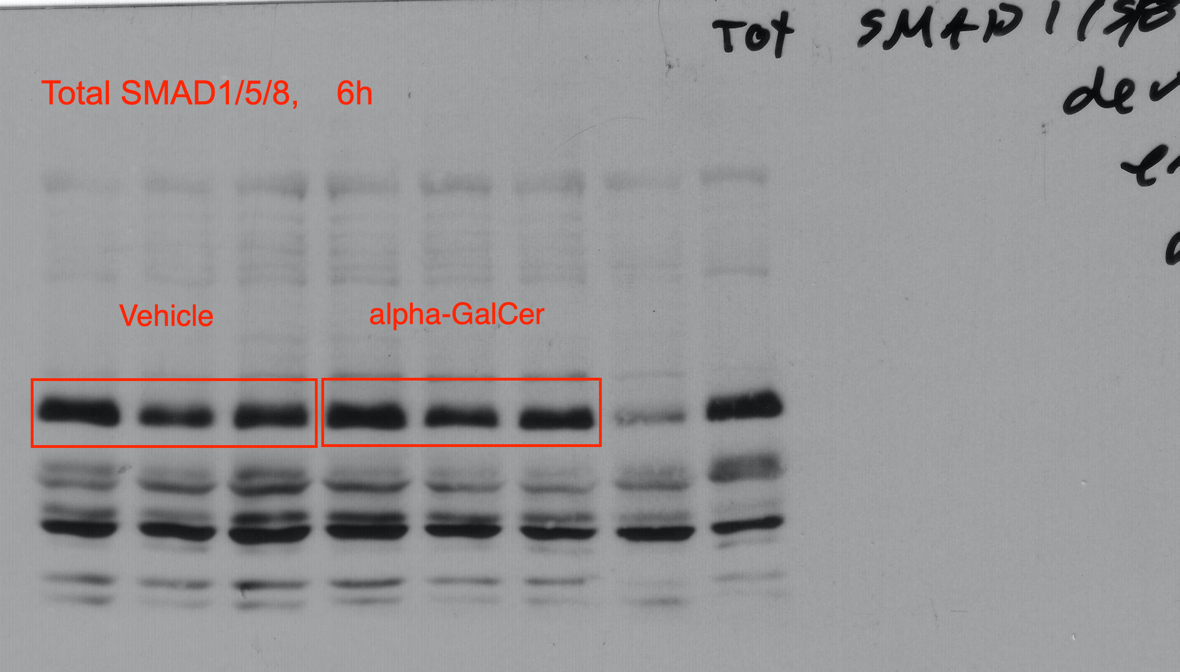
**

**pSMAD1/5/8 (upper blot) and Total SMAD1/5/8 (lower blot) 6 h post-treatment**

The last two lanes in both blots are controls representing liver samples extracted from mice kept on a standard diet (lane 8; control) and mice fed an iron-loading diet containing 2.5% carbonyl iron (lane 9), extracted two weeks after initiation of the diets. Lane 1 - marker (Precision Plus Protein™ Kaleidoscope™ Prestained Protein Standards, #1610395, from BioRad)

**
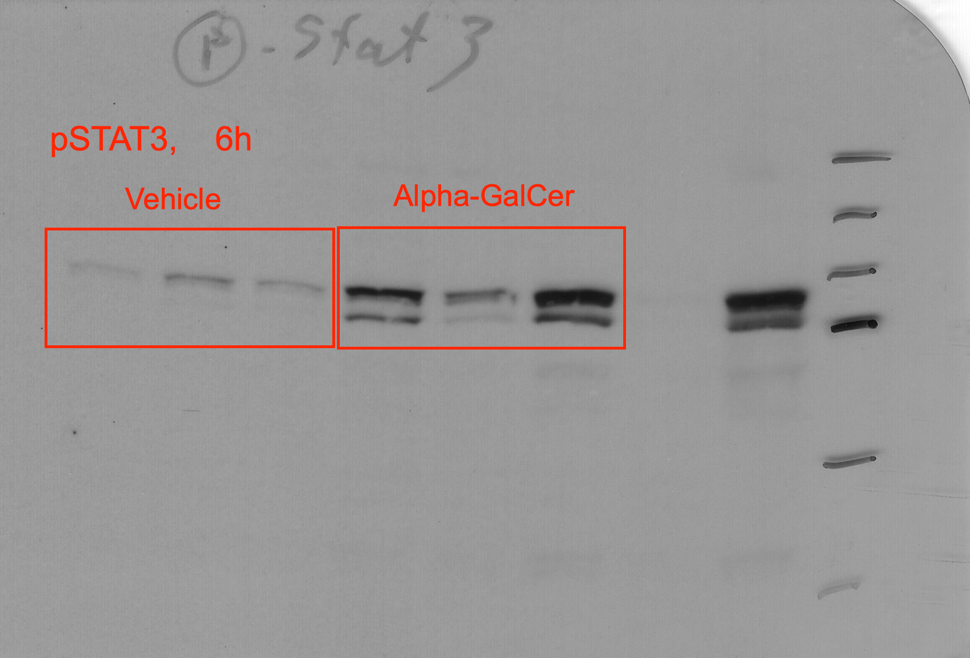
**

**
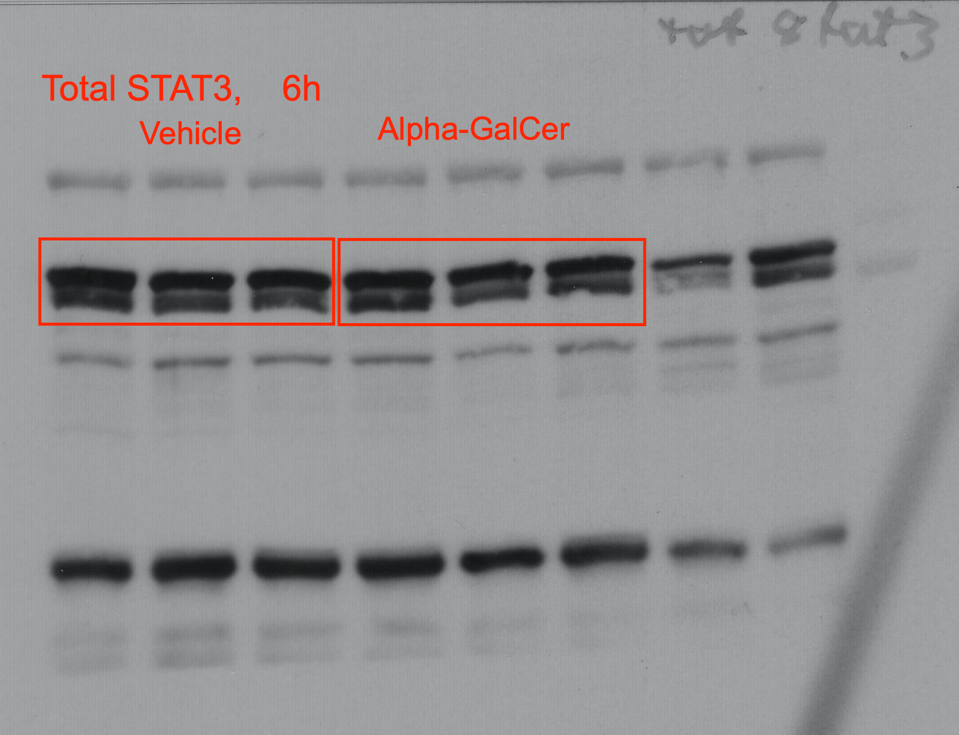
**

**pSTAT3 (upper blot) and Total STAT3 (lower blot) 6 h post-treatment**

The last two lanes in both blots are controls representing liver samples extracted from mice injected with PBS (lane 7; control) and mice injected with LPS (lane 8), extracted 6 h later. Lane 9 - marker (Precision Plus Protein™ Kaleidoscope™ Prestained Protein Standards, #1610395, from BioRad).


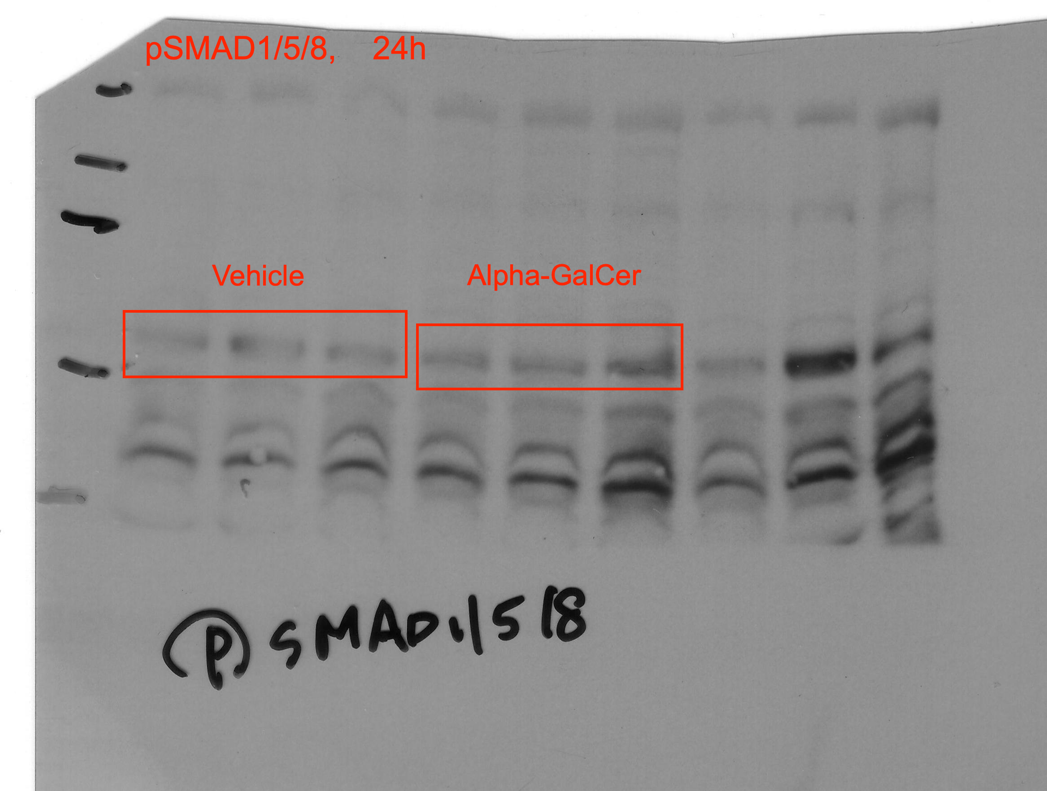


**
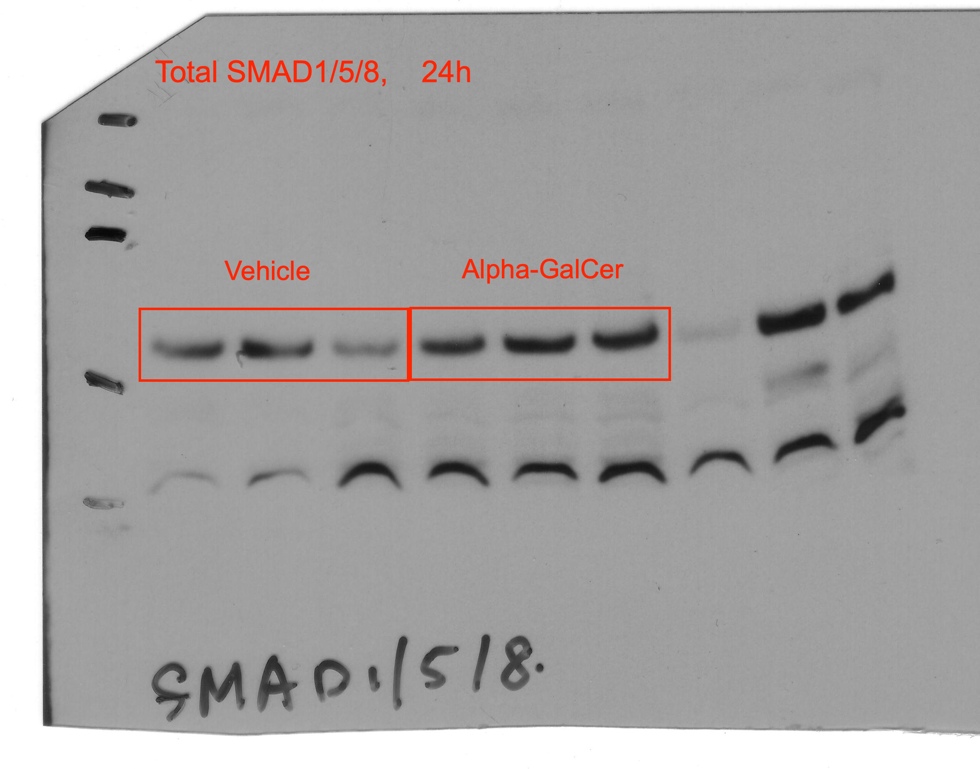
**

**pSMAD1/5/8 (upper blot) and Total SMAD1/5/8 (lower blot) 24 h post-treatment**

The last three lanes in both blots are controls representing liver samples extracted from mice kept on a standard diet (lane 8; control) and mice fed an iron-loading diet containing 2.5% carbonyl iron (lane 9), both of which were extracted two weeks after initiation of the diets, and mice injected with LPS extracted 6 h later (lane 10). Lane 1 - marker (Precision Plus Protein™ Kaleidoscope™ Prestained Protein Standards, #1610395, from BioRad)

**
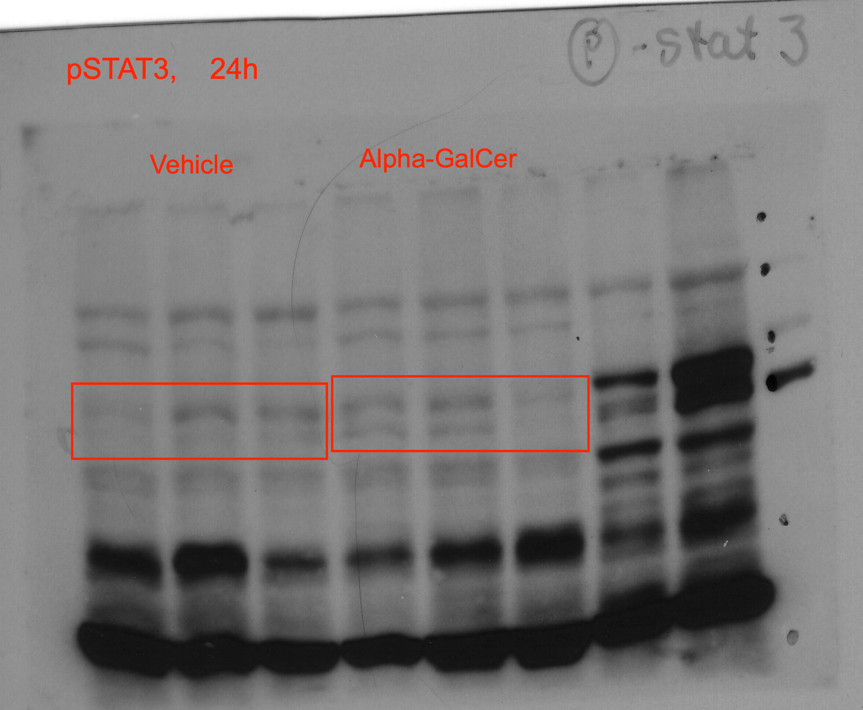
**

**
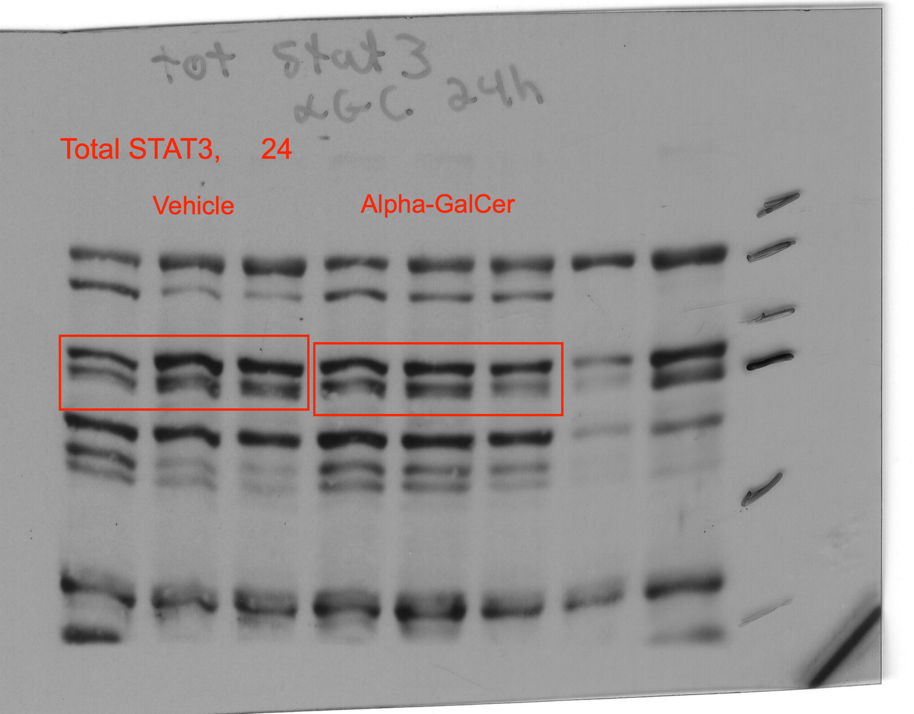
**

**pSTAT3 (upper blot) and Total STAT3 (lower blot) 24 h post-treatment**

The last two lanes before the marker (lane 9) in both blots are controls representing liver samples extracted from mice injected with PBS (lane 7; control) and mice injected with LPS (lane 8), extracted 6 h later. Lane 9 - marker (Precision Plus Protein™ Kaleidoscope™ Prestained Protein Standards, #1610395, from BioRad)

**Supplementary Methods**

**Supplementary Table 1. Primers used for qPCR analysis of mRNA levels**

| **Species** | **Gene** |  | **Sequence** |
| --- | --- | --- | --- |
| *Mus musculus* | ***Actb*** | Forward | TGTTACCAACTGGGACGACA |
|  |  | Reverse | GGTGTTGAAGGTCTCAAA |
|  | ***Hamp*** | Forward | CCTATCTCCATCAACAGATG |
|  |  | Reverse | AACAGATACCACACTGGGAA |
|  | ***Bmp6*** | Forward | GACAAGGAGTTCTCCCCACA |
|  |  | Reverse | CCAGCCAACCTTCTTCTGAG |
|  | ***Smad7*** | Forward | GGCATTCCTCGGAAGTCAAG |
|  |  | Reverse | CAGCCTGCAGTTGGTTTGAG |
|  | ***HPRT-1*** | Forward | CATTATGCCGAGGATTTGGA |
|  |  | Reverse | AATCCAGCAGGTCAGCAAAG |
|  | ***F4/80*** | Forward | CCCCAGTGTCCTTACAGAGTG |
|  |  | Reverse | GTGCCCAGAGTGGATGTCT |
|  | ***Clec4f*** | Forward | TGAGTGGAATAAAGAGCCTCCC |
|  |  | Reverse | TCATAGTCCCTAAGCCTCTGGA |

**Animals**

All animals received humane care according to the criteria outlined in the “Guide for the Care and Use of Laboratory Animals”. All mice used in the studies were females, and aged 6 weeks old at the beginning of the experiments. Mice were fed a standard diet (Teklad Global 18% protein rodent diet; Harlan (Envigo) Teklad Diets, Madison, WI). Mice were maintained at the Centre de recherche du CHUM (CRCHUM) and at the Maisonneuve-Rosemont Hospital Research Centre (CRHMR). Animals were kept at 22°C in humidity (65%) and lighting-controlled (12:12 light–dark cycle, light on at 07:00) rooms and had free access to chow and water. They were housed in specific pathogen-free (SPF) conditions in filter top cages layered with bedding material at 3–4 mice per cage. Nesting material as environmental enrichment was added to each cage and was changed every week. Treatments were performed during the light cycle.
